# Evolutionary radiations in the species-rich mountain genus *Saxifraga* L.

**DOI:** 10.1186/s12862-017-0967-2

**Published:** 2017-05-25

**Authors:** J. Ebersbach, J. Schnitzler, A. Favre, A.N. Muellner-Riehl

**Affiliations:** 1 Department of Molecular Evolution and Plant Systematics & Herbarium (LZ), Institute of Biology, Leipzig University, Johannisallee 21–23, D-04103 Leipzig, Germany; 2 German Centre for Integrative Biodiversity Research (iDiv) Halle-Jena-Leipzig, Deutscher Platz 5e, D-04103 Leipzig, Germany

**Keywords:** Evolutionary radiations, alpine habitats, *Saxifraga*, diversification rates, key innovations, Hengduan Mountains

## Abstract

**Background:**

A large number of taxa have undergone evolutionary radiations in mountainous areas, rendering alpine systems particularly suitable to study the extrinsic and intrinsic factors that have shaped diversification patterns in plants. The species-rich genus *Saxifraga* L. is widely distributed throughout the Northern Hemisphere, with high species numbers in the regions adjacent to the Qinghai-Tibet Plateau (QTP) in particular the Hengduan Mountains and the Himalayas. Using a dataset of 297 taxa (representing at least 60% of extant *Saxifraga* species), we explored the variation of infrageneric diversification rates. In addition, we used state-dependent speciation and extinction models to test the effects of geographic distribution in the Hengduan Mountains and the entire QTP region as well as of two morphological traits (cushion habit and specialized lime-secreting glands, so-called hydathodes) on the diversification of this genus.

**Results:**

We detected two to three rate shifts across the *Saxifraga* phylogeny and two of these shifts led to radiations within two large subclades of *Saxifraga,* sect. *Ciliatae* Haworth subsect. *Hirculoideae* Engl. & Irmsch. and sect. *Porphyrion* Tausch subsect. *Kabschia* Engl*.* GEOSSE analyses showed that presence in the Hengduan Mountains had a positive effect on diversification across *Saxifraga*. Influence of these mountains was strongest in *Saxifraga* sect. *Ciliatae* subsect. *Hirculoideae* given its pronounced distribution there, and thus the radiation in this group can be classified at least partially as geographic. In contrast, the evolution of the cushion life form and lime-secreting hydathodes had positive effects on diversification only in selected *Saxifraga* sections, including sect. *Porphyrion* subsect. *Kabschia*. We therefore argue that radiation in this group was likely adaptive.

**Conclusions:**

Our study underlines the complexity of processes and factors underpinning plant radiations: Even in closely related lineages occupying the same life zone, shifts in diversification are not necessarily governed by similar factors. In conclusion, alpine plant radiations result from a complex interaction among geographical settings and/or climatic modifications providing key opportunities for diversification as well as the evolution of key innovations.

**Electronic supplementary material:**

The online version of this article (doi:10.1186/s12862-017-0967-2) contains supplementary material, which is available to authorized users.

## Background

Evolutionary radiations resulting in exceptionally species-rich clades are shaping current patterns of biodiversity in the plant kingdom [1, 2]. Large numbers of evolutionary radiations have been recorded in alpine plant groups [3], and references therein, contributing to the high species diversity of vascular plants found in many mountain systems [4, 5]. In fact, alpine life zones around the world are proportionally more species-rich than expected by the area they occupy [6]. In addition, alpine habitats occur at all latitudes allowing for global comparisons [7], which renders them particularly attractive to study evolutionary radiations.

Mountain systems are influenced by several extrinsic factors and processes that have the potential to trigger plant radiations. First, geological processes can cause populations to become geographically isolated, fostering allopatric speciation [8, 9]. Additionally, orogenic activity creates new environmental space, which can be colonized by pre-adapted local or immigrant lineages, thereby providing key opportunities for diversification [10–12]. Yet, viewing mountain building as sole driver for diversification is likely an oversimplification [13]. Speciation and uplift usually occur on different time scales, and in fact, many mountain plant taxa have diversified long after the uplift of their respective mountain range was initiated, as shown for the species-rich areas surrounding the Qinghai-Tibet Plateau (QTP), [14, 15] and elsewhere [3]. Thus, other mechanisms are likely to have contributed to radiations in mountains. For example, several authors have demonstrated the importance of local or global climate modifications for alpine plant radiations [16, 17].

High elevation life zones are characterized by environmental conditions that limit plant growth, such as large diurnal temperature amplitudes or strong seasonality with severe winter frosts, extended snow cover and short growing seasons [18]. Thus, successful colonization, establishment and finally radiation in this life zone might depend on the evolution of traits that allow for adaptation and provide competitive advantages in alpine environments. Novel traits (morphological, physiological, etc.) that allow species to conquer new adaptive zones are referred to as key innovations and they are often regarded as an important first step in adaptive radiations as they allow for ecomorphological divergence and thus speciation [19, 20].

Key innovation potential of selected traits can be tested using phylogenetic trees: Positive effects of a particular trait should result in substantially higher diversification rates in groups possessing that trait. Several key innovations have been identified for different montane and alpine plants such as low specific leaf area in Ericaceae Juss. [21], cushion habit in *Androsace* L. [22], and fruit type, both in *Tripterospermum* Blume [23] and in Andean bellflowers [24].

The effects of orogeny, climate change, key opportunities and key innovations can interact with each other and may differ between mountain systems, which can result in regionally restricted radiations, so-called geographic radiations [19]. Studying alpine plant groups that occur in several mountain systems may therefore contribute to disentangling the factors triggering alpine radiations. In this study, we will identify rapidly evolving clades and investigate the potential drivers for these radiations in the broadly distributed herbaceous genus *Saxifraga*.

The large arctic-alpine genus *Saxifraga* (Saxifragaceae Juss.) comprises up to 500 species [25] and is widely distributed across the Northern Hemisphere. Species diversity is concentrated in the southern European mountain ranges, the Caucasus, the Arctic as well as the QTP region [26]. The Himalayas and the Hengduan Mountains boast particularly high species numbers with up to 75 species in the subnival belt of the Hengduan Mountains alone [27]. *Saxifraga* was confirmed to be monophyletic, provided the exclusion of *Micranthes* Haworth [28, 29]. The genus comprises at least 13 sections with species of widely differing morphology and varying levels of species richness, from the monotypic section *Saxifragella* (Engl.) Gornall & Zhang to the very large section *Ciliatae* (175 species) [26]. For example, species of section *Porphyrion* (90–112 species) are mostly cushion plants not taller than a few centimetres, whereas *Irregulares* Haworth species (10–20 species) are erect, with large, petiolate basal leaves, and reaching up to 40 cm in height [26, 30]. Several sections within *Saxifraga* have radiated within relatively short geological time-frames [31, 32], but the underlying factors behind these radiations have not been studied in detail. For these reasons, *Saxifraga* is an ideally suited study system to investigate patterns and processes connected to alpine radiations. Integrating phylogenetic, biogeographical, and morphological information, we here present a multifaceted approach to studying diversification in *Saxifraga*. We will focus on the following questions: (1) Do diversification rate shifts explain differences in clade size within *Saxifraga*? (2) Do diversification rates within *Saxifraga* vary with geographic distribution? (3) Did the evolution of the cushion life form and lime-secreting hydathodes, both of which are potential key innovations in alpine habitats, affect diversification in *Saxifraga*?

## Results

### Diversification rate patterns in *Saxifraga*

**Fig. 1:**
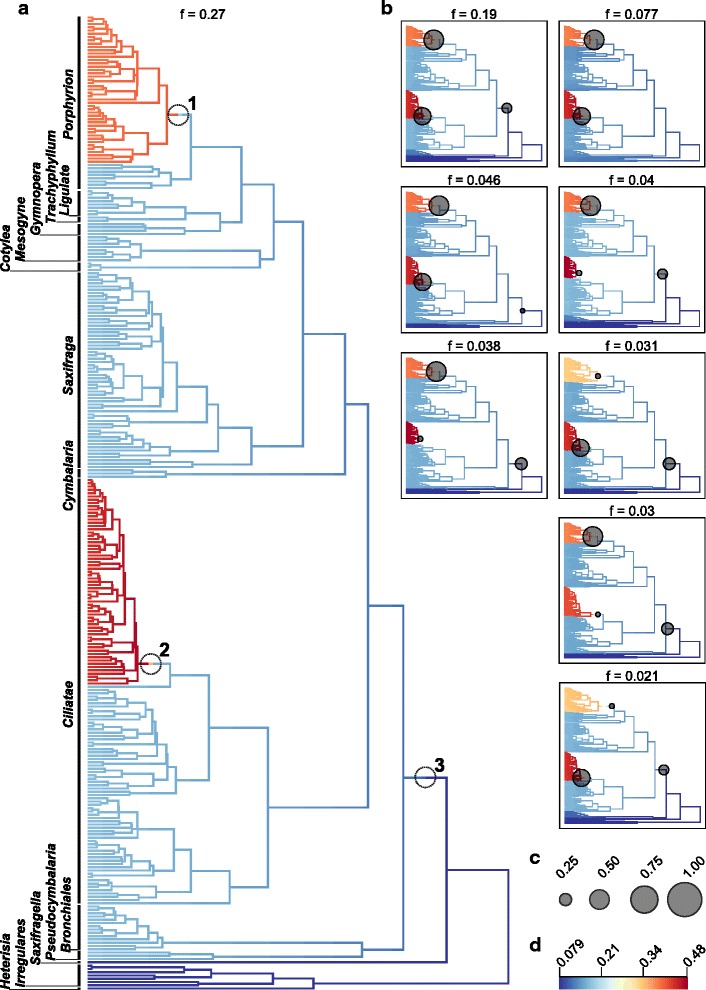
Diversification dynamics in *Saxifraga*. **a** Best shift scenario according to BAMM. Dashed circles indicate diversification rate shifts. *Saxifraga* sections are indicated according to Tkach et al. [26] and Gornall et al. [33]. **b** The eight next likely shift scenarios. **c** Size reference for marginal shift probabilities for each shift in B. **d** Colour ramp for diversification rates in species per million years. Results presented here are from the analysis with γ = 0.5 and minimum sampling fractions per section. Results from additional BAMM analyses were concordant with those presented here and are summarized in Additional file 1

Independent of prior choice and assumed total species numbers, BAMM identified two to three rate shifts within *Saxifraga* (Fig. 1, Additional file 1). Two of these shifts were consistently placed within the same *Saxifraga* clades across all analyses. The first one (from here on referred to as rate shift 1) was located within sect. *Porphyrion* subsect. *Kabschia* *sensu* Tkach et al. [26], which originated and radiated in Europe (European Alps, Caucasus Mountains) and includes one subgroup that colonized the QTP region and diversified there*.* The second one (shift 2) was located within a clade that contains members of sect. *Ciliatae* subsect. *Hirculoideae*. This clade is the largest subclade of the species-rich section *Ciliatae*, which originated and radiated in the QTP region [31–33]. A third rate shift (shift 3) was present in some scenarios and placed in varying locations close to the root of the *Saxifraga* phylogeny, most often on the node separating sections *Heterisia* (A. M. Johnson) Small, *Irregulares* and *Saxifragella* (see Fig. 1) from the rest of the genus.

Analyses with BayesRate confirmed the presence of three diversification rate shifts within *Saxifraga* (Table 1). Regarding overall rate regimes, the model with three distinct diversification rates had the highest marginal likelihood: one rate shared by both clades affected by rate shifts 1 and 2, one rate shared by sections *Heterisia*, *Irregulares*, *Saxifragella* and *Pseudocymbalaria* Zhmylev (group H + I + S + P), and one rate for the rest of *Saxifraga* (Bayes factor (BF) min: 11.66, BF max 9.61; Table 1). Additionally, models specifying pure birth processes performed considerably better than those with birth-death processes, with the exception of group H + I + S + P for which both processes were almost equally likely (Table 1). Rate estimates did not differ substantially between BAMM and BayesRate (Additional file 2). While BayesRate generally produced slightly higher net diversification rate estimates (Additional file 2), the considerable overlap of the 95% HPD intervals indicates an overall agreement between the two approaches. Importantly, the differences between *Ciliatae* subsect. *Hirculoideae* and the other clades were found to be very similar (e.g., an increase over the background rate of 2.6- and 2.4-fold, respectively).

**Table 1:** Comparison of different diversification rate scenarios for *Saxifraga* using BayesRate

Total no. of shifts	*Ciliatae* subsect. *Hirculoideae*	*Porphyrion* subsect. *Kabschia*	Group H + I + S + P	Back-ground	min		max	
					LogL	BF	LogL	BF
3	PB*		PB	PB	-665.42	-	-693.57	-
3	PB*		BD	PB	-666.13	1.42	−694.82	2.50
3	PB*		BD	BD	-668.67	6.50	−697.40	7.66
3	BD*		BD	BD	-670.55	10.25	−698.69	10.23
2	PB*		-	PB	-671.25	11.66	−698.38	9.61
2	PB*		-	BD	-673.20	15.55	−700.35	13.56
2	PB	PB	-	PB	-674.16	17.48	−701.91	16.67
2	BD*		-	BD	-675.16	19.49	−702.43	17.72
2	BD	BD	-	BD	-679.17	27.50	−706.92	26.70
1	PB	-	-	PB	-682.72	34.59	−708.13	29.10
1	BD	-	-	BD	-686.32	41.80	−711.20	35.26
1	-	PB	-	PB	-700.92	71.00	−725.73	64.32
1	-	PB	-	PB	-703.61	76.37	−728.10	69.05
0	-	-	-	PB	-722.36	113.88	−748.80	110.46
0	-	-	-	BD	-723.53	116.22	−749.73	112.31

Various scenarios for shifts in three *Saxifraga* clades under pure birth (PB) and birth-death (BD) processes were tested. Asterisks indicate scenarios in which selected clades were constrained to have the same net diversification rates. Log marginal likelihoods (LogL) and results from Bayes factor (BF) tests are given for analyses with minimum and maximum species

Diversification rate estimates were very similar between minimum and maximum species number analyses (Table 2). Under pure birth processes, net diversification rates in clades *Ciliatae* subsect. *Hirculoideae* and *Porphyrion* subsect. *Kabschia* were about 2.5 times higher than the background rate (net diversification rates min, max: *Hirculoideae* and *Kabschia* clade 0.455, 0.471 species/Myr; background rate 0.171, 0.184 species/Myr; Table 2). Sections *Heterisia*, *Irregulares*, *Saxifragella* and *Pseudocymbalaria* on the other hand diversified at a rate that was ca. 3 times lower than the *Saxifraga* background rate (min, max: 0.054, 0.071 species/Myr).

**Table 2:** Net diversification rate estimates

	min mean [HPD]	max mean [HPD]
*Ciliatae* subsect. *Hirculoideae*	0.455 [0.349–0.575]	0.471 [0.357–0.593]
*Porphyrion* subsect. *Kabschia*	0.455 [0.349–0.575]	0.471 [0.357–0.593]
Group H + I + S + P	0.054 [0.020–0.090]	0.071 [0.032–0.116]
Background rate	0.171 [0.133–0.211]	0.184 [0.144–0.226]

Mean net diversification (speciation - extinction) rates and 95% highest posterior density (HPD) intervals for the different *Saxifraga* clades from the BayesRate analysis

### Geography-associated diversification

**Fig. 2:**
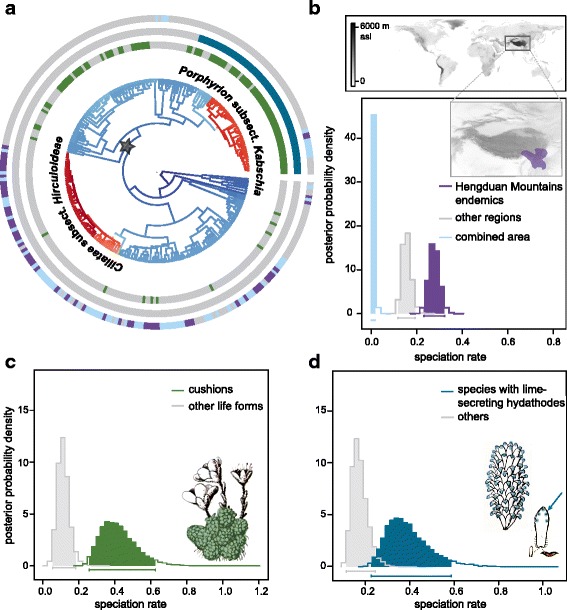
State-dependent diversification in *Saxifraga*. **a** *Saxifraga* phylogeny with estimated diversification rate regimes according to BAMM (Fig. 1, warmer colours indicate higher rates). Phylogenetic distributions of species’ traits are indicated in green (cushion habit) and blue (lime-secreting hydathodes), the geographical distribution (presence in the Hengduan Mountains) is shown in purple. **b** Speciation rates in the Hengduan Mountain hotspot vs. other regions. **c** Speciation rates of cushion saxifrages vs. other life forms. Analysis was constrained to parts of the *Saxifraga* phylogeny indicated by the star in **a** Drawing depicts habit of *S. lilacina* [87], cushion indicated by green shading. **d** Speciation rates of lineages carrying lime-secreting hydathodes vs. others. The analysis was constrained to parts of the *Saxifraga* phylogeny indicated by the star in **a** Drawings depict stem of *S. imbricata*, with lime-secreting hydathodes on each leaf tip and a single cauline leaf of *S. lilacina* bearing five lime-secreting hydathodes [87]

The GEOSSE model with the lowest overall AIC value specified a pure birth process in the Hengduan Mountains region (area A) and differential speciation, extinction and transition rates between areas (Additional file 3.1). Two similar models, also with differential speciation and transition rates between areas, also had good model fit (ΔAIC < 6; Additional file 3.1). Initial parameter estimates using maximum likelihood inference of these three best scoring models were highly congruent (Additional file 3.2) and we decided to use the best overall model to run MCMC parameter estimation. Net diversification rates were ca. 3.5 times higher in the Hengduan Mountains region than in the rest of the world (Fig. 2, Table 3). The empirical ΔAIC for the Hengduan Mountain dataset was well separated from the distribution of simulated ΔAIC values indicating a robust signal in the empirical data for region-dependent diversification (Additional file 4).

**Table 3:** Parameter estimates for best scoring GEOSSE model for state-dependent diversification of *Saxifraga* in the Hengduan Mountains region

	λ mean [HPD]	μ mean [HPD]	d mean [HPD]	r mean [HPD]
Hengduan Mountains	0.277	–	0.464	0.277
	[0.231–0.321]		[0.316–0.623]	[0.231–0.321]
Other areas	0.156	0.076	0.004	0.080
	[0.118–0.193]	[0.021–0.133]	[0.001–0.008]	[0.049–0.11]
Joined area	0.006			
	[0.000–0.018]			

Mean parameter estimates and 95% highest probability density (HPD) intervals are given for speciation rates (λ), extinction rate (μ), transition rates to joined area (d) and net diversification rates (r; λ – μ) of *Saxifraga* in the Hengduan Mountains region

Analyses regarding the entire QTP region yielded similar results with respect to the most likely diversification model and parameter estimates (Additional files 5.1 and 2). However, there was no substantial difference in model fit between simulated and empirical data suggesting that asymmetrical transition rates alone, not differential diversification, could have led to the observed distribution pattern (Additional file 4).

### Tests for key innovations

When tested across the entire *Saxifraga* phylogeny, neither the evolution of lime-secreting hydathodes nor the cushion exhibited significant patterns of differential speciation or extinction rates. However, as pointed out by Beaulieu & Donoghue [34] and Beaulieu & O’Meara [35], it can be problematic to use BiSSE to investigate the effects of certain traits on phylogenies with multiple rate shifts. Both of the traits that we tested are limited to specific clades of *Saxifraga* (Fig. 2). Importantly, they are almost entirely absent in *Ciliatae* and in *Ciliatae* subsect. *Hirculoideae* for which rate shift 2 was detected*.* Restricting the analysis to a smaller part of the tree comprising sections *Cymbalaria* Griseb., *Saxifraga*, *Cotylea* Tausch, *Mesogyne* Sternb*.*, *Gymnopera* D. Don, *Trachyphyllum* (Gaudin) W. D. J. Koch, *Ligulatae* Haworth and *Porphyrion* yielded a different result: Plants with lime-secreting hydathodes and cushion saxifrages had substantially higher diversification rates (Fig. 2, Table 4). In addition, trait simulations showed that the empirical ΔAIC were substantially different from simulated ΔAIC values revealing a strong signal for differential diversification rates under these two traits for this part of the *Saxifraga* phylogeny (Additional file 4).

**Table 4:** Parameter estimates for best scoring models for state-dependent diversification for cushion life form and lime-secreting hydathodes in *Saxifraga*

	λ mean [HPD]	μ mean [HPD]	q mean [HPD]	r mean [HPD]
Cushions	0.420	0.219	0.056	0.201
	[0.247–0.614]	[0–0.452]	[0.024–0. 096]	[0.094–0.312]
Other life forms	0.114	0.065	0.055	0.048
	[0.051–0.179]	[0–0.188]	[0.002–0.119]	[−0.074–0.143]
Hydathodes	0.393	0.159	0.003	0.234
	[0.226–0.587]	[0–0.395]	[0–0.009]	[0.118–0.356]
Non–hydathodes	0.173	0.063	0.006	0.110
	[0.113–0.244]	[0–0.150]	[0–0.015]	[0.057–0.163]

Mean parameter estimates and 95% highest probability density (HPD) intervals are given for speciation rates (λ), extinction rates (μ); transition rates (q) and net diversification rates (r; λ – μ)

## Discussion

As a species-rich group with an affinity to high altitude habitats, *Saxifraga* is well suited to study underlying patterns and factors related to alpine radiations and the diversification process in general. Our study shows that multiple rate shifts led to radiations within *Saxifraga* and that specific combinations of extrinsic and intrinsic factors drove these radiations. To our knowledge, this is the most extensive study on diversification rates with regard to the species-rich, yet still poorly understood, alpine areas surrounding the QTP to date.

### Diversification rate patterns in *Saxifraga*

We found a slight increase in the inferred number of rate shifts when increasing the hyperprior on the expected number of rate shifts (Additional file 1A), but prior choice did not affect model selection (scenario with maximum posterior probability; Additional file 1B & C). This was further corroborated in a recent study by Mitchell & Rabosky [36]. While both model selection via Bayes factors, as well as pure-birth models of diversification would also be available in BAMM, BayesRate additionally allows for speciation and extinction rates to be linked between clades. In our case, this revealed that speciation and extinction rates of clades *Ciliatae* subsect. *Hirculoideae* and *Porphyrion* subsect. *Kabschia* were not significantly different (i.e., a model with same rates was preferred over one with independent rates for both clades).

We found evidence for three distinct diversification rate shifts within *Saxifraga*. Two of these rate shifts led to substantially increased diversification rates within *Saxifraga* sections *Ciliatae* and *Porphyrion* which had previously been suggested as having experienced rapid radiations [31, 32]. Therefore, diversification rate shifts at least partially explain clade size differences in *Saxifraga*.

Only a few diversification rate estimates have been published for other taxa with pronounced radiations in the QTP region. The rate estimates reported here (mean estimates min, max: 0.455, 0.486 species/Myr) were similar to those found in other alpine radiations from the QTP region such as in *Gentiana* L. (0.37 species/Myr) [37] and in several subgenera of *Delphinium* L. (subg. *Delphinastrum* (DC.) Peterm. and *Oligophyllon* Dimitrova 0.37–0.81 species/Myr, subg. *Aconitum* 0.42–1.11 species/Myr, all assuming low extinction) [38]. In contrast, diversification rates were very low (0.066 species/Myr) in *Rhododendron* L. despite the taxon being famously species-rich in the QTP region [21]. In addition, the rate estimates reported here do not match the exceptionally high rates that have been reported for several Andean plant groups (e.g., *Lupinus* L.: 1.56–5.21 species/Myr [39]; *Gentianella* Moench 1.48–3.21 species/Myr [40]) despite the fact that the Andes and the Hengduan Mountains share several environmental features (e.g., young age, high physiographic heterogeneity). This suggests that comparisons of alpine radiations across the globe might also need to incorporate regional dynamics (e.g., geologic and glaciation history, biogeographic connectivity).

An additional rate shift was present towards the root of the phylogeny, separating sections *Heterisia*, *Irregulares, Saxifragella* and *Pseudocymbalaria* (group H + I + S + P) with very low net diversification rates from the rest of *Saxifraga*. Pure birth and birth-death processes were almost equally likely in this clade, so this depauperate clade may result from very low speciation, relatively high extinction or a combination thereof. In general, pure birth processes had superior model fit in *Saxifraga* and this matches very low extinction rate estimates that have been found for several other alpine plant groups [21, 37]. However, extinction rates are difficult to estimate from molecular phylogenies and rate estimates are often low with large confidence intervals [35, 41, 42] but see [43].

### Drivers of diversification within *Saxifraga*

Similar to *Saxifraga*, multiple infrageneric rate shifts yielding substantial increases in diversification rates were shown in other species-rich alpine or subalpine genera such as *Rhododendron* L. [21], *Lupinus* L. [39] and *Hypericum* L. [16], suggesting that some plant groups might be particularly prone to radiate. This could be due to intrinsic or extrinsic factors or factor combinations that are shared by all infrageneric clades involved in these radiations. In *Saxifraga*, this seems unlikely as clades affected by rate shifts are morphologically and phylogenetically distinct with contrasting distribution patterns and biogeographic histories [26, 31]. Rather, it appears plausible that each rate shift was triggered by a specific combination of intrinsic and extrinsic factors including geographical distribution and key innovations as will be discussed in the next section.

#### Influence of the Hengduan Mountains region on diversification

Occurrence in the Hengduan Mountains region had a positive effect on *Saxifraga* diversification. Section *Ciliatae* subsect. *Hirculoideae* affected by rate shift 2 is distributed almost entirely in the QTP region with a large proportion of species occurring in the Hengduan Mountains [30, 44]. While some *Ciliatae* taxa recently colonized other areas including East Asia, Northern Asia and even North America, they did not diversify in those areas [31]. Thus, local key opportunities in the Hengduan Mountains region at the time of diversification may have triggered the diversification rate increase in this group. While rapid radiations have been documented for several plant groups distributed in these mountains [3], ours is the first study to formally compare diversification rates of taxa distributed in the Hengduan Mountains region with those of close relatives of other areas.

The Hengduan Mountains stand out from other mountain ranges in the QTP region due to several characteristics. In contrast to the plateau, parts of which had reached 4000 m altitude as early as 40 MY ago [14, 45] and the Himalayas, which were uplifted to significant altitudes during the Miocene [15], the Hengduan Mountains are considered to be relatively young (Miocene, late Pliocene) [46, 47]. These different geological histories have led to strongly contrasting biogeographic patterns throughout the region. While recent *in situ* diversification was disproportionally more important for the species assembly in the Hengduan Mountains, Himalayan biodiversity was largely influenced by immigration [48]. The Hengduan Mountains and the eastern Himalayas have been under the influence of the monsoon system (i.e., greater summer rainfall) since their orogeny, whereas the QTP proper and the western Himalayas are almost entirely beyond the reach of the summer monsoon. In addition, extinctions during Pleistocene glaciations were likely low due to north-south orientation of the valleys of the Hengduan Mountains region [48]. Finally, the Hengduan Mountains have been described as highly heterogeneous in terms of topography, featuring deeply dissected landscapes with steep elevational gradients [3].

Recently, the interplay of high physiographic heterogeneity and episodes of rapid climate oscillations (promoting the "species pump" effect) has been highlighted as a potential driver of alpine diversification [19, 49]. Acting together, these two factors may enhance diversification in mountains by repeatedly modifying dispersal barriers, thus promoting allopatric speciation while buffering extinction through the availability of nearby refugia resulting in a large number of species with an island-like distribution and a high chance of survival. Among others, global climate oscillations have been reconstructed for the Early Pliocene as well as for the Pleistocene [50, 51]. This time corresponds roughly to the period that was suggested as origin of the majority of species within subsect. *Hirculoideae* (<5 Myr ago) [31]. Therefore, both high habitat heterogeneity and climate oscillations were present during the early stages of the radiation of *Saxifraga* in the Hengduan Mountains. Extant *Saxifraga* distribution patterns fit the expected results of the "species pump" effect and long-term survival: There are at least 35 *Saxifraga* species endemic to the subnival belt of the Hengduan Mountains, many of them with highly restricted distribution areas [30, 44].

However, while subsect. *Hirculoideae* showed a substantial shift in its diversification rate, the remaining subgroups of sect. *Ciliatae* that also predominantly occur in the QTP region (and particularly in the Hengduan Mountains) did not. This strongly suggests that the presence in these mountains is not sufficient to explain the diversification rate pattern in this section and that geographic radiations might require additional factors other than extrinsic key opportunities. Subsection *Hirculoideae* is not characterized by any obvious morphological or physiological traits that could serve as clade-specific key innovations. Another driver that has been found to be associated to plant radiations and shifts in diversification rates are whole genome duplications [52]. A survey of polyploidy in the flora of the Hengduan Mountains showed that, contrary to expectations, the frequency of polyploidy in the flora was low (22%) [53]. For *Saxifraga*, the authors found that the proportion of polyploid species was higher than average (44%) but still lower that what would have been expected judging from other high mountain floras (45–85%) [53]. However, chromosome numbers were only available for nine *Saxifraga* species distributed in the Hengduan Mountains, so further work will be required to examine this pattern in more detail. Regarding hybridization, another mechanism often connected to rapid speciation in plants, we were only able to find one report of a potential trace of such an event in *S. egregia* of sect. *Ciliatae* [54], however more data is needed to clarify this. Finally, niche shifts or changes in niche width could also be an important factor involved in diversification rate shifts, as demonstrated, for instance, by Matuszak et al. [23] and Favre et al. [37] who found that increased diversification rates corresponded to niche shifts in two species-rich genera of Gentianinae G. Don (i.e., *Gentiana* and *Tripterospermum*)*.* For *Saxifraga*, Rubio de Casas et al. [55] showed that the cliff and rock face habitat was most common but that a switch to tundra habitats (which exhibited high within-habitat diversification) occurred within a large unresolved clade containing species of sections *Ciliatae*, *Saxifraga* and *Porphyrion*. Since the exact location of this shift within the phylogeny remained unclear, ecological niches will have to be studied in more detail for these sections to investigate this pattern more closely.

#### Key Innovations in alpine environments

*Saxifraga* rate shift 1 was observed in *Porphyrion* subsect. *Kabschia,* which is largely distributed in European and Asian mountain systems, with only one subclade occurring in the QTP region, where the subsection is more diverse in the Himalayas than in the Hengduan Mountains [56]. Due to this wide and uneven distribution pattern, it is unlikely that key opportunities connected to geographic distribution have triggered the rate shift in this group. Rather, morphological traits likely acted as key innovations causing the observed rate shift in *Porphyrion* subsect. *Kabschia*. First, the cushion life form, which is widespread throughout the genus, was associated with differential rates of diversification in certain clades of *Saxifraga*. Cushion plants are widespread in arctic and alpine habitats and this life form has been shown to be an adaptation to cold climates through reduction in water loss and desiccation [57]. Similar to *Saxifraga*, the cushion life form was found to foster lineage diversification in the species-rich alpine genus *Androsace* [22, 58]. The results derived from both genera corroborate the findings of Boucher et al. [57] who showed that the cushion life form arose more than 100 times independently across Angiosperms and concluded that it is likely to be a convergent key innovation for occupancy of extremely cold environments.

Rather than a single key innovation driving radiations, it has been suggested that several sequentially acquired traits may act together as drivers of lineage diversification [59]. Beside the cushion habit, lime-secreting hydathodes, which are restricted to sections *Ligulatae* and *Porphyrion*, were also associated with higher diversification rates in some clades of *Saxifraga*. Hydathodes, in general, are associated with guttation, or water discharge from the leaf interior, and occur widely in *Saxifraga* [60]. Lime-secreting hydathodes which are morphologically distinct from simple hydathodes serve as a means for excreting excess calcium salts leaving characteristic white encrustations on the leaves [61]. This mechanism is particularly interesting considering that many *Saxifraga* species, in particular those of sections *Porphyrion*, *Ligulatae*, *Trachyphyllum* and *Gymnopera*, occur in habitats with little soil, including cliff ledges and rock crevices, and display strong substrate specificity with regards to calcareous or siliceous substrates. Conti et al. [61] suggested that lime-secreting hydathodes represent the derived state of hydathodes in *Saxifraga* and current phylogenetic placement of the aforementioned sections suggests that they evolved either once in the ancestor of these four sections and were subsequently lost twice (in sections *Mesogyne* and *Trachyphyllum*), or less likely, that lime-secretion evolved twice independently in *Porphyrion* and in *Ligulatae*. Following the first scenario, it would be likely that the ancestor of these four sections was calcicole. However, at this point substrate preference data are not complete for species of sect. *Porphyrion*. While there is a general trend that species possessing lime-secreting hydathodes occur on base-rich, calcareous soils or rock, there are several exceptions to this pattern: Several species with lime-secreting pores are not bound to base-rich substrates (e.g., *S. juniperifolia*, *S. cotyledon*, *S. florulenta*) and species that do not possess lime-secreting hydathodes can occur alongside others that do on calcareous soils (e.g., *S. exarata* subsp. *moschata*) [60]. Thus, this mechanism and its implications clearly deserve closer attention, in particular considering its positive correlation with diversification in *Saxifraga* as shown in this study.

In general, it is important to point out that while we have achieved substantial taxon coverage of *Saxifraga*, there are some species missing from our dataset, in particular from sections for which rate shifts were inferred (e.g., section *Irregulares*: sampling proportion: 0.35–0.7%, section *Porphyrion*: 0.47–0.58%, Additional file 6). Sampling bias can affect the detection of rate shifts as well as the location of identified shifts [62, 63] and caution when interpreting the results is therefore advised. However, we took this into account as thoroughly as possible by supplying section-specific sampling proportions during all analyses and running minimum and maximum species number analyses. Furthermore, most sections of *Saxifraga* are morphologically distinct and molecular work so far confirmed the current taxonomic delineation [26]. Thus, it is unlikely that these missing taxa would affect our results.

## Conclusion

Our study highlights the complexity of plant radiations. Even in closely related lineages occupying the same life zone, shifts in diversification rates are not necessarily governed by the same factors. In particular, radiation within one subgroup of *Saxifraga* (section *Ciliatae* subsect. *Hirculoideae*) was driven by extrinsic opportunities associated with the Hengduan Mountains region as well as additional factors, possibly niche shifts. A second infrageneric radiation (in sect. *Porphyrion* subsect. *Kabschia*) was likely independent of these processes. Instead, the cushion habit and the acquisition of lime-secreting hydathodes emerged as likely key innovations indicating that the radiation in this clade might have been adaptive (*sensu* Simões et al. [19]). The case of *Saxifraga* thus shows that alpine plant radiations have complex underlying causes, which are to be viewed in both, geographical and biotic contexts. Future studies of (alpine) plant radiations should therefore attempt to identify combinations of intrinsic and extrinsic factors relevant to the diversification process.

## Methods

### Dataset

For this study, we used the same Saxifragaceae data set as in Ebersbach et al. [31], consisting of 420 taxa and an aligned length of 4225 bp of markers ITS, *trn*L–*trn*F, and *mat*K. Prior to analyses, we modified the posterior distribution of post-burnin BEAST trees from Ebersbach et al. [31] by removing all taxa not belonging to *Saxifraga* as well as several duplicate taxa. In addition, three taxa (*S. haplophylloides*, *S. diffusicallosa* and *S. maxionggouensis*) from the dataset of Gao et al. [32] were added to all trees using the bind.tip function of the *phytools* package [64] in R [65]. This function allows to add tips to a phylogeny while rescaling the tree to conserve its ultrametric properties. According to Gao et al. [32] these three taxa are all part of subsect. *Hirculoideae* which form a well-defined subclade of *Saxifraga* section *Ciliatae*, but their exact position is unclear due to low resolution. We therefore placed these taxa randomly within that subclade (also using randomly generated node heights) to mirror the existing uncertainty. The final dataset consisted of 297 *Saxifraga* taxa. Analyses were run on the maximum clade credibility (MCC) tree generated by TreeAnnotator v1.8.2 [66] as well as on a set of 100 trees randomly sampled from all trees in the posterior distribution.

### Diversification rates

We used BAMM (v.2.5.0) [67] and the R package *BAMMtools* [68] as well as the programme BayesRate (v.1.6.3, [43]) to assess diversification rate heterogeneity across the *Saxifraga* phylogeny. BAMM identifies distinct configurations of rate shifts without the need for an *a priori* specification of their number and location. Any sampling bias is likely to affect the accuracy of rate estimates [69], so we specified section-specific sampling fractions to account for incomplete taxon sampling. However, given that published species numbers for some sections differ widely (Additional file 6, e.g., [26, 30, 70, 71]), we calculated sampling fractions for minimum and maximum published species numbers per section and ran all analyses with both values.

We ran four MCMC chains with 20 million generations each, saving the output every 1000th generation. The first 10% of samples were discarded as burn-in and the effective sample sizes for the number of shifts and the log likelihood were calculated to assess convergence. It has been suggested that the likelihood function of BAMM might be incorrect and that the programme suffers from strong prior sensitivity and unreliable rate estimates [72]. However, Rabosky et al. (in press) [73] recently reported that they were unable to reproduce the prior sensitivity and that the shortcomings indicated by Moore et al. [72] were likely due to a misapplication of the programme and poorly designed test data. We performed two additional analyses to address these issues: First, we ran the analyses under a range of settings for the prior distribution on the number of rate shifts (γ = 10, 2, 1, 0.5, 0.1) to assess the potential prior sensitivity. Second, we used the programme BayesRate to further evaluate the various rate shift scenarios. BayesRate was specifically designed for hypothesis-based testing of diversification regimes (i.e., rate shifts through time, rate differences between particular clades, etc.) across a given phylogeny or set of phylogenies. Importantly, BayesRate employs a different likelihood function [42] than BAMM, and thus provides an independent test of the diversification model (number of rate shifts) and the associated rates. Finally, BayesRate allows for a straightforward incorporation of phylogenetic (topological and temporal) uncertainty, while there is currently no direct way of accounting for phylogenetic uncertainty in BAMM. We used BayesRate to test the most likely number of rate regimes and to compare the likelihood of pure birth (yule) and birth-death via thermodynamic integration. After identifying the best-fit model via a Bayes factor test [74], we estimated the model parameters across all 100 randomly sampled phylogenetic trees to account for phylogenetic uncertainty.

### Key innovations

We tested the effect of two potential key innovations (cushion habit and the presence of lime-secreting hydathodes) on *Saxifraga* diversification. Trait information was assembled from several floras as well as specific *Saxifraga* literature [30, 60, 75–77]. We supplied state-specific sampling proportions to account for missing species and sampling bias (sampling proportions: cushion plants: 0.8, non-cushions 0.5; plants with lime-secreting hydathodes: 0.85; plants without lime-secreting hydathodes: 0.55). To asses trait-associated diversification, we fitted the Multiple State Speciation and Extinction (MuSSE) model [78] on 100 phylogenetic trees in a Bayesian framework (as outlined in [79], script available at https://github.com/dsilvestro/mcmc-diversitree). MuSSE is a multistate extension of the BiSSE (Binary State Speciation and Extinction) model [80], which simultaneously estimates the rates of speciation (λ) and extinction (μ) under two character states (0 and 1) as well as transition rates between these states (q_01_ and q_10_).

Recently, serious concerns about the statistical power and interpretations of state-dependent speciation and extinction (SSE) models have been raised [81–83]. The statistical power of BISSE and other SSE models is relatively low when using small phylogenies with less than 300 tips and/or trait states that are unevenly represented in the phylogeny (high tip ratio). Furthermore, SSE models have been shown to be affected by high Type 1 error rates that stem from not accounting for independent shifts in character states that are not associated to diversification *per se* [83]. In order to account for this, we employed a simulation scheme suggested by Rabosky & Goldberg [83] exploiting the Maximum Likelihood framework of BiSSE in the R package *diversitree* [78]. The empirically estimated mean transition rates q_10_ and q_01_ were used to simulate 100 sets of traits across the *Saxifraga* phylogeny using the R package *phytools* [64]. The simulated traits were then used to fit two models each, the unconstrained diversification model (λ_0_ ≠ λ_1_, μ_0_ ≠ μ_1_, q_0_ ≠ q_1_) and the best scoring model for each trait. We used the distribution of ΔAIC values (difference in AIC between both models) to investigate the discriminative power of these models and compared this with the results from our empirical analysis.

### Geographical distribution

GeoSSE, another member of the SSE model class, is specifically designed to investigate the association of geographical distribution patterns with lineage diversification [84]. It estimates rates of speciation in the target area (area A) and the remaining distribution (area B) as well as in the joint range (area AB). Range contraction is modelled as local extinction (AB -> B, AB -> A) while transition rates between each area and the joint area (A -> AB, B -> AB) correspond to range expansion. We used GeoSSE as implemented in the R package *diversitree* [78] to test for an association between diversification in *Saxifraga* and occurrence in the Hengduan Mountains region. Distribution data were retrieved from various floras (30, 60, 75, 76; sampling proportion of *Saxifraga* from Hengduan Mountains region: 0.5, in other areas: 0.7, widespread species in combined area: 0.7). In order to test the robustness of our results we performed an additional analysis testing for an effect of occurrence in the QTP region in general, here broadly defined as the QTP itself, the Hengduan Mountains, the Himalayas, the Karakorum Range, Pamir, the Tianshan, and the Altai Mountains (sampling proportion of *Saxifraga* from QTP region: 0.65, in other areas: 0.8, widespread species in combined area: 0.9). Area delineation of the Hengduan Mountains region was done in accordance with Boufford [85]. In total, we compared 36 constrained GeoSSE models (e.g., equal speciation, extinction, and/or transition rates) to a fully unconstrained model (differential speciation, extinction and transition rates) using the AIC and Akaike weights [86]. Consecutively, parameter estimation was performed via an MCMC analysis of 50,000 generations for the model with the lowest AIC. The first 500 steps were discarded as burn-in and trait simulations were run as described above.

## Additional files

Additional file 1: Results of prior sensitivity tests and species number comparisons in BAMM (PDF 1143 kb)

Additional file 2: Diversification rate estimates for *Saxifraga* from BayesRate and BAMM (DOCX 32 kb)

Additional file 3: 1. Best scoring GEOSSE models for state-dependent diversification of *Saxifraga* in Hengduan Mountains. 2. Initial parameter estimates of GEOSSE models for state-dependent diversification of *Saxifraga* in Hengduan Mountains (DOCX 39 kb)

Additional file 4: Results for model fitting of simulated traits in *Saxifraga (PDF 416 kb)*

Additional file 5: 1. Best scoring GEOSSE models for state-dependent diversification of *Saxifraga* in QTP region. 2. Parameter estimates for best scoring GEOSSE model for state-dependent diversification of *Saxifraga* in QTP region (DOCX 35 kb)

Additional file 6: Minimum and maximum species numbers for all *Saxifraga* sections (DOCX 29 kb)

## Abbreviations

QTP: Qinghai-Tibet Plateau
H + I + S + P: Clade consisting of sections *Heterisia*, *Irregulares*, *Saxifragella*, *Pseudocymbalaria*
BF: Bayes factor
PB: Pure birth (or Yule) diversification process
BD: Birth-death diversification process
HPD: Highest posterior density
SSE model: State-dependent speciation and extinction model
