# Supplementary material for: Evolutionary radiations in the species-rich mountain genus Saxifraga L
Source: BMC Evol Biol. 2017 May 25;17:119. doi: 10.1186/s12862-017-0967-2 (PMC5445344; doi:10.1186/s12862-017-0967-2)
Supplement: Supplementary file 1 — Results of prior sensitivity tests and species number comparisons in BAMM (PDF 1143 kb) [file 12862_2017_967_MOESM1_ESM.pdf]

Additional file 1. Summary of BAMM analyses.

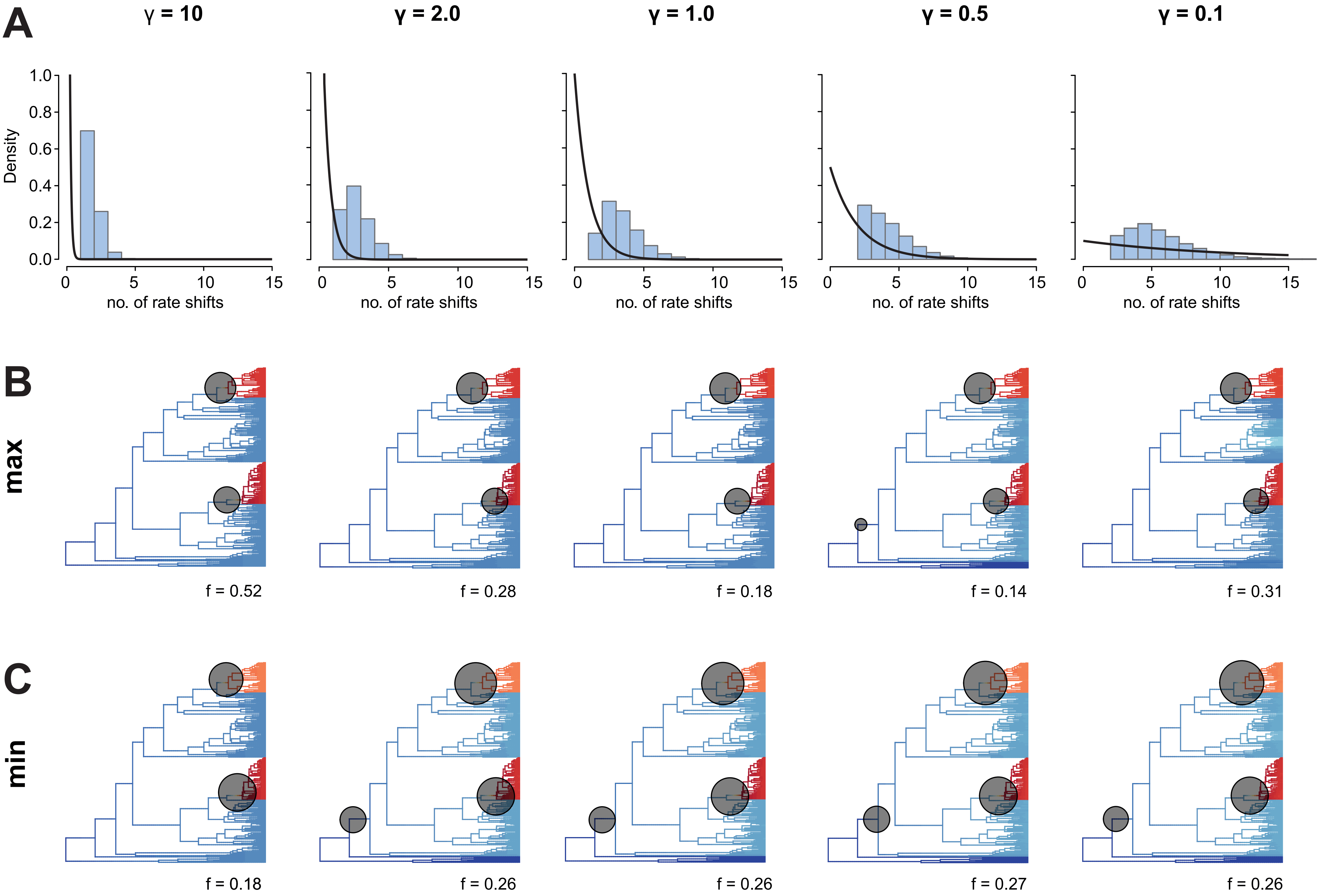

We analyzed the *Saxifraga* dataset under a range of prior values for the expected number of diversification rate shifts in accordance with Moore et al. 2016 (columns; γ = 10, 2, 1, 0.5, 0.1) and specified minimum and maximum section-specific sampling fractions. A: Comparisons of prior (black line) and posterior (blue bars) distribution for the number of rate shifts within *Saxifraga*. B: Corresponding shift scenario with maximum posterior probability (f) from BAMM for each prior value for maximum species number analyses. Warmer colors (red) indicate higher diversification rates. C: Corresponding shift scenario with maximum posterior probability (f) from BAMM for each prior value for minimum species number analyses. Warmer colors (red) indicate higher diversification rates.
