# Supplementary material for: Evolutionary radiations in the species-rich mountain genus Saxifraga L
Source: BMC Evol Biol. 2017 May 25;17:119. doi: 10.1186/s12862-017-0967-2 (PMC5445344; doi:10.1186/s12862-017-0967-2)
Supplement: Supplementary file 2 — Diversification rate estimates for Saxifraga from BayesRate and BAMM (DOCX 32 kb) [file 12862_2017_967_MOESM2_ESM.docx]

**Additional file 2**

**Diversification rate estimates for *Saxifraga* from BayesRate and BAMM.**

|  | **BayesRate** | | **BAMM** | |
| --- | --- | --- | --- | --- |
|  | **min** | **max** | **min** | **max** |
| **Speciation rate** | **mean [HPD]** | **mean [HPD]** | **mean [HPD]** | **mean [HPD]** |
| *Ciliatae* subsect. *Hirculoideae* | 0.455 **[**0.349‒0.575**]** | 0.471 **[**0.357‒0.593**]** | 0.409 **[**0.251‒0.497**]** | 0.409 **[**0.329‒0.503**]** |
| *Porphyrion* subsect. *Kabschia* | 0.455 **[**0.349‒0.575**]** | 0.471 **[**0.357‒0.593**]** | 0.371 **[**0.251‒0.497**]** | 0.423 **[**0.315‒0.563**]** |
| H+I+S+P | 0.054 **[**0.020‒0.090**]** | 0.071 **[**0.032‒0.116**]** | 0.081 **[**0.033‒0.148**]** | 0.123 **[**0.052‒0.186**]** |
| Background rate | 0.171 **[**0.133‒0.211**]** | 0.184 **[**0.144‒0.226**]** | 0.158 **[**0.136‒0.184**]** | 0.164 **[**0.140‒0.191**]** |
| **Extinction rate** | **mean [HPD]** | **mean [HPD]** | **mean [HPD]** | **mean [HPD]** |
| *Ciliatae* subsect. *Hirculoideae* | - | - | 0.050 **[**0.004‒0.144**]** | 0.052 **[**0.004‒0.147**]** |
| *Porphyrion* subsect. *Kabschia* |  |  | 0.076 **[**0.004‒0.219**]** | 0.096 **[**0.006‒0.267**]** |
| H+I+S+P |  |  | 0.044 **[**0.002‒0.135**]** | 0.047 **[**0.002‒0.167**]** |
| Background rate |  |  | 0.014 **[**0.002‒0.037**]** | 0.016 **[**0.002‒0.044**]** |
| **Net diversification rate** | **mean [HPD]** | **mean [HPD]** | **mean [HPD]** | **mean [HPD]** |
| *Ciliatae* subsect. *Hirculoideae* | 0.455 **[**0.349‒0.575**]** | 0.471 **[**0.357‒0.593**]** | 0.358 **[**0.279‒0.438**]** | 0.357 **[**0.277‒0.438**]** |
| *Porphyrion* subsect. *Kabschia* | 0.455 **[**0.349‒0.575**]** | 0.471 **[**0.357‒0.593**]** | 0.294 **[**0.185‒0.387**]** | 0.327 **[**0.228‒0.419**]** |
| H+I+S+P | 0.054 **[**0.020‒0.090**]** | 0.071 **[**0.032‒0.116**]** | 0.037 **[**-0.014‒0.115**]** | 0.076 **[**-0.001‒0.142**]** |
| Background rate | 0.171 **[**0.133‒0.211**]** | 0.184 **[**0.144‒0.226**]** | 0.144 **[**0.122‒0.167**]** | 0.147 **[**0.123‒0.171**]** |

Mean rate estimates and 95% highest posterior density (HPD) intervals are given for speciation rates, extinction rates and net diversification rates.
