# Supplementary material for: Evolutionary radiations in the species-rich mountain genus Saxifraga L
Source: BMC Evol Biol. 2017 May 25;17:119. doi: 10.1186/s12862-017-0967-2 (PMC5445344; doi:10.1186/s12862-017-0967-2)
Supplement: Supplementary file 3 — 1. Best scoring GEOSSE models for state-dependent diversification of Saxifraga in Hengduan Mountains. 2. Initial parameter estimates of GEOSSE models for state-dependent diversification of Saxifraga in Hengduan Mountains (DOCX 39 kb) [file 12862_2017_967_MOESM3_ESM.docx]

**Additional file 3.1**

**Best-scoring GEOSSE models for state-dependent diversification of *Saxifraga* in Hengduan Mountains.**

| No. | speciation | extinction | PB | transition | Df | lnLiK | AIC | ΔAIC | weights |
| --- | --- | --- | --- | --- | --- | --- | --- | --- | --- |
| 1 | λ_A_≠λ_B_ , λ_A_≠λ_AB_, λ_B_≠λ_AB_ | µ_A_≠µ_B_ | A | q_A_≠q_B_ | 6 | -1021.2 | 2054.3 | 0 | 0.670 |
| 2 | λ_A_≠λ_B_ , λ_A_≠λ_AB_, λ_B_≠λ_AB_ | µ_A_≠µ_B_ | ‒ | q_A_≠q_B_ | 7 | -1021.2 | 2056.3 | 2 | 0.246 |
| 3 | λ_A_≠λ_B_ , λ_A_≠λ_AB_, λ_B_≠λ_AB_ | µ_A_=µ_B_ | A,B | q_A_≠q_B_ | 5 | -1024.8 | 2059.6 | 5.3 | 0.047 |
| 4 | λ_A_≠λ_B_ , λ_A_≠λ_AB_, λ_B_≠λ_AB_ | µ_A_=µ_B_ | ‒ | q_A_≠q_B_ | 6 | -1024.8 | 2061.6 | 7.3 | 0.017 |
| 5 | λ_A_≠λ_B_ , λ_A_≠λ_AB_, λ_B_≠λ_AB_ | µ_A_≠ | B | q_A_≠q_B_ | 6 | -1024.8 | 2061.6 | 7.3 | 0.017 |

Legend: Parameters of models with more than 0.05 relative probability are presented for region A (Hengduan Mountains), B (remaining distribution areas) or AB (combined distribution areas A and B). Model numbers correspond to Additional file 3.2. PB: Pure‒Birth process. Degrees of freedom (Df), Log Likelihoods (lnLik), Akaike Information Criterion (AIC) [1], difference in AIC to best model (ΔAIC) and Akaike weights [2] are given.

**Additional file 3.2**

**Initial parameters estimates of GEOSSE models for state-dependent diversification of *Saxifraga* in Hengduan Mountains using Maximum Likelihood function of diversitree.**

| No. | λ_A_ | λ_B_ | λ_AB_ | µ_A_ | µ_B_ | q_A_ | q_B_ |
| --- | --- | --- | --- | --- | --- | --- | --- |
| 1 | 2.75E-01 | 1.52E-01 | 0.00E+00 | - | 7.27E-02 | 4.50E-01 | 3.80E-03 |
| 2 | 2.75E-01 | 1.52E-01 | 5.00E-06 | 0.00E+00 | 7.15E-02 | 4.49E-01 | 3.85E-03 |
| 3 | 2.80E-01 | 1.16E-01 | 1.00E-07 | - | - | 3.80E-01 | 5.68E-03 |
| 4 | 2.80E-01 | 1.16E-01 | 1.20E-06 | 0.00E+00 | 0.00E+00 | 3.80E-01 | 5.68E-03 |
| 5 | 2.80E-01 | 1.16E-01 | 8.80E-06 | 4.90E-06 | - | 3.80E-01 | 5.69E-03 |

Legend: Model numbers correspond to Additional file 3.1. Speciation rate (λ), extinction rate (µ) and transition (q) rate estimates for region A (Hengduan Mountains), B (remaining distribution areas) or AB (combined distribution areas A and B) are given.

**References**

1. Akaike H. Information theory and an extension of the maximum likelihood principle. In: Petrov BN, Caski F, editors. Proceedings of the Second International Symposium on Information Theory. Budapest: Akademiai Kiado; 1973. p. 267-281.

2. Akaike H. On the Likelihood of a Time Series Model. Statistician 1978;27:217.
