# Supplementary material for: Evolutionary radiations in the species-rich mountain genus Saxifraga L
Source: BMC Evol Biol. 2017 May 25;17:119. doi: 10.1186/s12862-017-0967-2 (PMC5445344; doi:10.1186/s12862-017-0967-2)
Supplement: Supplementary file 4 — Results for model fitting of simulated traits in Saxifraga (PDF 416 kb) [file 12862_2017_967_MOESM4_ESM.pdf]

Additional File 4: Results for model fitting of simulated traits in *Saxifraga*.

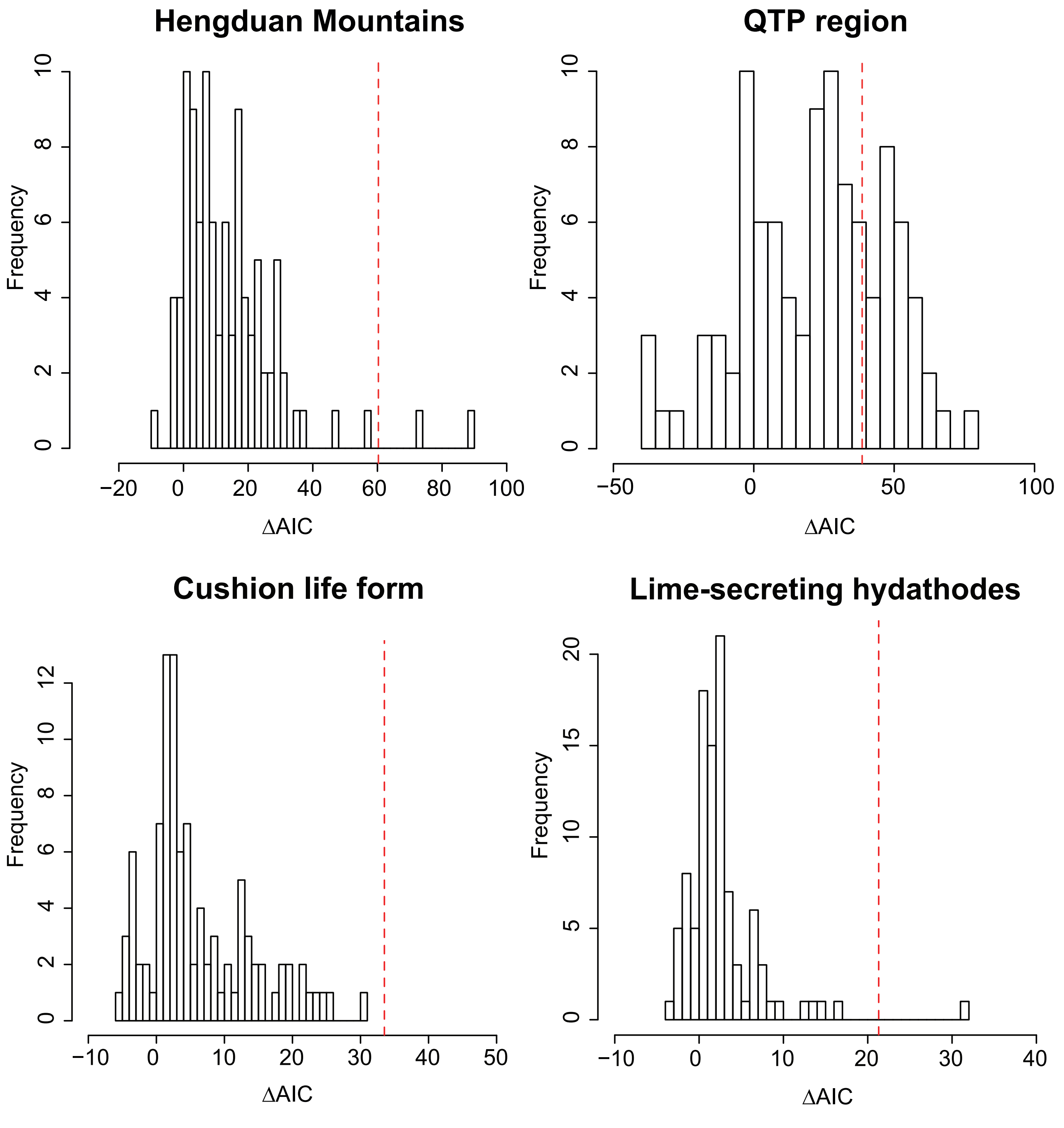

Empirically estimated transition rates for geographical distribution in Hengduan Mountains (A) or QTP region (B), evolution of cushion life form (C) and lime-secreting hydathodes (D) were used to simulate sets of 100 traits each. These were fitted to two models: the best state-dependent diversification model (according to AIC values for all tested GEOSSE and BiSSE models) and a model with equal speciation and extinction in all areas/trait states (no state-dependent diversification). Histograms show differences in model fit as ΔAIC for simulated data. Red vertical lines indicate Δ AIC for empirical data. Empirical Δ AIC values that fall to the right of the simulated Δ AIC distribution indicate that the emprical data have stronger support for state-dependent diversification (larger difference in model fit) and that transition rates alone do not explain the observed trait distribution across the phylogeny.
