# Supplementary material for: Evolutionary radiations in the species-rich mountain genus Saxifraga L
Source: BMC Evol Biol. 2017 May 25;17:119. doi: 10.1186/s12862-017-0967-2 (PMC5445344; doi:10.1186/s12862-017-0967-2)
Supplement: Supplementary file 5 — 1. Best scoring GEOSSE models for state-dependent diversification of Saxifraga in QTP region. 2. Parameter estimates for best scoring GEOSSE model for state-dependent diversification of Saxifraga in QTP region (DOCX 35 kb) [file 12862_2017_967_MOESM5_ESM.docx]

**Additional file 5.1**

**Best scoring model parameters for GEOSSE models for state-dependent diversification of *Saxifraga* in QTP region.**

| speciation | extinction | PB | transition | Df | lnLik | AIC | ΔAIC | weights |
| --- | --- | --- | --- | --- | --- | --- | --- | --- |
| λ_A_≠λ_B_ , λ_A_≠λ_AB_, λ_B_≠λ_AB_ | µ_A_≠µ_B_ | A | q_A_≠q_B_ | 6 | -939.87 | 1891.8 | 0 | 0.459 |
| λ_A_≠λ_B_ , λ_A_≠λ_AB_, λ_B_≠λ_AB_ | µ_A_=µ_B_ | ‒ | q_A_≠q_B_ | 7 | -939.87 | 1893.8 | 2 | 0.169 |
| λ_A_≠λ_B_ , λ_A_≠λ_AB_, λ_B=_λ_AB_ | µ_A_≠µ_B_ | A | q_A_≠q_B_ | 5 | -941.95 | 1893.9 | 2.1 | 0.161 |
| λ_A_≠λ_B_ , λ_A_≠λ_AB_, λ_B=_λ_AB_ | µ_A_≠µ_B_ | ‒ | q_A_≠q_B_ | 6 | -941.95 | 1895.9 | 4.1 | 0.059 |

Legend: Parameters of models with more than 0.05 relative probability are presented for region A (QTP region), B (remaining distribution areas) or AB (combined distribution areas A and B). PB: Pure‒Birth process. Degrees of freedom (Df), Log Likelihoods (lnLik), Akaike Information Criterion (AIC) [1], difference in AIC to best model (ΔAIC) and Akaike weights [2] are given.

**Additional file 5.2**

**Parameter estimates for best scoring GEOSSE model for state-dependent diversification of *Saxifraga* in QTP region.**

|  | **λ** | **µ** | **d** | **r** |
| --- | --- | --- | --- | --- |
| **QTP region** | 0.292 [0.211‒0.292] | ‒ | 0.535 [0.02‒0.062] | 0.292 [0.211‒0.292] |
| **other areas** | 0.129 [0.118‒0.2] | 0.049 [0.019‒0.137] | 0.006 [0.002‒0.009] | 0.08 [0.049‒0.111] |
| **joined area** | 0.004 [0‒0.159] |  |  |  |

Legend: Mean parameter estimates and 95% highest probability density (HPD) intervals are given for speciation rates (λ) , extinction rate (µ); transition rates to joined area (d) and net diversification rates (r; λ ‒ µ).

**References**

1. Akaike H. Information theory and an extension of the maximum likelihood principle. In: Petrov BN, Caski F, editors. Proceedings of the Second International Symposium on Information Theory. Budapest: Akademiai Kiado; 1973. p. 267-281.

2. Akaike H. On the Likelihood of a Time Series Model. Statistician 1978;27:217.
