# Supplementary material for: Evolutionary radiations in the species-rich mountain genus Saxifraga L
Source: BMC Evol Biol. 2017 May 25;17:119. doi: 10.1186/s12862-017-0967-2 (PMC5445344; doi:10.1186/s12862-017-0967-2)
Supplement: Supplementary file 6 — Minimum and maximum species numbers for all Saxifraga sections (DOCX 29 kb) [file 12862_2017_967_MOESM6_ESM.docx]

**Additional file 6**

Minimum and maximum species numbers for all Saxifraga sections and corresponding sampling proportions used in diversification rate analyses.

| **section** | **published**  **species numbers** | **sampled** | **sampling proportion**  **min** | **sampling proportion**  **max** |
| --- | --- | --- | --- | --- |
| *Porphyrion* | 90‒112^[1,3]^ | 53 | 0.58 | 0.47 |
| *Ligulatae* | 8^[1]^ | 8 | 1 | 1 |
| *Trachyphyllum* | 2^[1]^ | 2 | 1 | 1 |
| *Gymnopera* | 4^[1]^ | 4 | 1 | 1 |
| *Mesogyne* | 8‒11^[1,3]^ | 8 | 1 | 0.73 |
| *Cotylea* | 2‒3^[1,4]^ | 2 | 1 | 1 |
| *Saxifraga* | 70‒85^[1]^ | 60 | 0.86 | 0.71 |
| *Cymbalaria* | 3^[1]^ | 3 | 1 | 1 |
| *Ciliatae* | 175^[3]^ | 130 | 0.74 | 0.74 |
| *Bronchiales* | 14‒20^[1,2]^ | 14 | 1 | 0.7 |
| *Pseudocymbalaria* | 3^[1]^ | 3 | 1 | 1 |
| *Saxifragella* | 1^[1]^ | 1 | 1 | 1 |
| *Irregulares* | 10‒20^[1,3]^ | 7 | 0.7 | 0.35 |
| *Heteresia* | 1^[1]^ | 1 | 1 | 1 |

References

1. Tkach N, Röser M, Miehe G, Muellner-Riehl AN, Ebersbach J, Favre A, Hoffmann MH. Molecular phylogenetics, morphology and a revised classification of the complex genus *Saxifraga* (Saxifragaceae). Taxon 2015:1159–87. doi:10.12705/646.4.

2. DeChaine EG, Anderson SA, McNew JM, Wendling BM. On the evolutionary and biogeographic history of *Saxifraga* sect. *Trachyphyllum* (Gaud.) Koch (Saxifragaceae Juss.). PLoS ONE 2013;8:e69814. doi:10.1371/journal.pone.0069814.

3. Pan J, Gornall RJ, Ohba H. *Saxifraga. In:* Wu C, Raven PH, editors. Flora of China: Brassicaceae through Saxifragaceae. Beijing, St. Louis: Science Press; Missouri Botanical Garden Press; 2001. p. 280–344.

4. Zhang Z, Chen S, Gornall RJ. Morphology and anatomy of the exine in *Saxifraga* (Saxifragaceae). Phytotaxa 2015;212:105. doi:10.11646/phytotaxa.212.2.1.
